# Physiological Adaptation to Different Heavy Metal Stress in Seedlings of Halophyte *Suaeda liaotungensis*

**DOI:** 10.3390/biology14030260

**Published:** 2025-03-05

**Authors:** Jieqiong Song, Xiaoqi Cao, Ruixuan An, Haoran Ding, Wen Wang, Yahan Zhou, Chunyan Wu, Yizihan Cao, Hongfei Wang, Changping Li, Qiuli Li

**Affiliations:** Key Laboratory of Plant Biotechnology of Liaoning Province, School of Life Sciences, Liaoning Normal University, Dalian 116081, China; songjieqiong2014@126.com (J.S.); cxq18340604311@163.com (X.C.); 19932021575@163.com (R.A.); 15898185529@163.com (H.D.); 13898902537@163.com (W.W.); 15718626632@163.com (Y.Z.); wcy18342365209@163.com (C.W.); 13390071535@163.com (Y.C.); whfei2008@126.com (H.W.); licp022@lnnu.edu.cn (C.L.)

**Keywords:** heavy metal pollution, *Suaeda liaotungensis*, dimorphic seeds, seedling growth, physiological response

## Abstract

Soil contamination with heavy metals is a worldwide environmental issue that impacts plant growth and human health. This study is the first to investigate the tolerance and physiological response mechanism of *Suaeda liaotungensis* seedlings to heavy metal stress. The results exhibited that the toxicity degree of Pb, Cd, Cu, and Zn to *Suaeda liaotungensis* seedlings was highest for Cd and lowest for Pb. Heavy metal stress increased H_2_O_2_ levels in seedlings, thereby aggravating lipid peroxidation of the cell membrane and consequently increasing MDA content. Meanwhile, the SOD and CAT activities in seedlings increased under heavy metal stress, whereas POD activity decreased consistently under Cd and Zn stress. The soluble sugars and proline content in seedlings also showed an increasing trend under heavy metal stress. Furthermore, the tolerance in the seedlings from black seeds to Pb and Cd stress was improved by enhancing SOD and CAT activities and accumulating proline. However, the tolerance in the seedlings from brown seeds to Cu stress was improved by increasing CAT activity as well as accumulating soluble sugar and proline content. The results reveal the response mechanism of *Suaeda liaotungensis* seedlings to heavy metal stress and provide the basis for utilizing *Suaeda liaotungensis* to improve heavy metal-contaminated saline soil.

## Simple Summary

In this study, the physiological response mechanism of the halophyte *Suaeda liaotungensis* to different heavy metal stress was discussed. We observed the growth and physiological changes in seedlings from dimorphic seeds of *Suaeda liaotungensis* under heavy metal stress. The toxicity degree of Pb, Cd, Cu, and Zn to the seedlings was Pb < Zn < Cu < Cd. This species reduces the toxicity of heavy metals through osmotic regulation and an antioxidant system. In addition, the seedlings from black seeds have strong tolerance to Pb and Cd stress, while those of brown seeds have strong tolerance to Cu stress. This study fills the research gap of studying this species under heavy metal stress. This study provides an important theoretical basis for this species being a potential candidate for repairing coastal saline soil contaminated by heavy metals.

## 1. Introduction

In recent years, some areas of coastal wetlands have faced many problems such as diverse soil pollutants, high concentrations of heavy metals and distinct regional variations due to factors like seawater erosion, oil and gas extraction, and wastewater discharge [1,2,3]. Wetlands are the most complex ecosystem with biodiversity in nature, and they are also an effective reservoir of heavy metal pollutants. These pollutants are not easily degraded by microorganisms and can be released into the upper water body through certain physical, chemical, and biological actions, making wetlands an important secondary pollution source. Heavy metal pollutants have characteristics such as a wide array of sources, difficult degradation, and easy accumulation, which have a great negative impact on ecosystem health [4]. The mass fraction of seven heavy metals (Hg, Pb, Cd, As, Cr, Cu, Zn) in the typical salt marsh soil of the Liaohe Estuary shows an increasing trend in spatial distribution, and the pollution degree is Cd > As > Cu > Zn > Pb > Cr > Hg [5]. Cu and Zn are regarded as essential micronutrients for plants, and their presence in excess is also potentially toxic, while Hg, Pb, Cd, and As are extremely poisonous [6,7,8]. Among them, Cd has a long biological half-life and can lead to mutations, cytotoxicity, and carcinogenicity [9]. The source of Cd pollution mainly comes from industrial emissions and agricultural activities such as the application of fertilizer [10]. Pb and Zn are released through mining activities and steel smelting [11,12]. Cu is released from numerous industrial processes and the overuse of fungicides, while fungicides containing copper are widely applied to prevent crop diseases [13]. The Cu and Zn content in the sediments of the Liaohe Estuary wetland is high, with the highest content of 108.2 and 207.7 μg/g, respectively [14]. Heavy metal contaminants such as Cu, Ni, Pb, and Zn are also detected in the lower reaches of the Haihe River wetland [15]. In the Bhaluka district of Bangladesh, the Cd and Pb levels in more than half of wetland water do not meet World Health Organization drinking water standards [16].

The accumulation of heavy metals in soil may have negative impacts on soil microbial communities, plant communities, and biogeochemical cycles, thus affecting biodiversity. Heavy metal toxicity may inhibit the growth and reproduction of soil microorganisms, lead to changes in the microbial community structure, and further affect soil biochemical processes, such as the decomposition of organic matter and nutrient cycling [17]. The accumulation of heavy metals such as Cd, Cu, Zn, Cr, and Ni in soil changes the chemical properties of the soil, such as the pH value and redox potential, thereby affecting the migration and transformation process of elements in soil [18]. These heavy metals may circulate in the soil–water–atmosphere system through leaching, adsorption, and desorption, thereby affecting biogeochemical cycles. Heavy metal toxicity also affects the morphological, physiological, and biochemical processes of plants, including seed germination, seedling growth and development, photosynthesis, plant water balance, and different metabolic processes essential for plant survival and growth [19,20]. Some studies have revealed that heavy metals can affect seed germination and seedling development by increasing free radical formation, disrupting cell osmotic regulation and reducing proteolytic activity [21,22,23]. The combined stress of Zn, Cu, and high salt can significantly inhibit seed germination in *Suaeda salsa* [24]. The plant height, fresh weight, and dry weight of *Salicornia europaea* have decreased significantly with the increase in Cd and Pb concentrations [25]. Cd toxicity affects chlorophyll content, photosynthetic rates, and intracellular CO_2_ concentrations [26]. Cu and Zn also reduce chlorophyll content as a result of the decreased photosynthetic efficiency in *Thalassia hemprichii* [27]. In addition, heavy metal (Pb, Cd, Cu, and Zn) toxicity inhibits plant growth and development mainly by imposing several constraints, such as oxidative stress, secondary osmotic stress, and ionic toxicity [28]. However, halophytes have mechanisms including regulating osmotic regulation, antioxidant systems, and using chelation to deimmobilize heavy metals to mitigate the toxicity of heavy metals, allowing them to survive heavy metal stresses [29]. Hence, halophytes have considerable potential in the phytoremediation and plant stability of soil contaminated with heavy metals [29,30].

*Suaeda liaotungensis* is a coastal halophyte that grows both on inland saline soil and intertidal zones near the estuary of the Liaohe River. It has strong stress tolerance, especially salt tolerance. Brown seeds have stronger salt tolerance than black seeds during germination in *S. liaotungensis*, and the germination rate of brown seeds still reaches 19% under 1400 mM NaCl treatment [31,32]. Meanwhile, the fresh and dry weights of plants (shoot, root, and total) were promoted obviously under 200 mM NaCl treatment [32]. In addition, *Suaeda* plants possess the capability to absorb heavy metals [28]. The efficiency in the bioaccumulation of metals in *Suaeda salsa* shows that it has a high capacity to accumulate Cd, Cu, Zn, and Pb from soil [33]. *S. salsa* can accumulate more Cr, Cu, Pb, and As, when compared to other halophytes, such as *Phragmites australis*, *Spartina alterniflora*, and *Typha orientalis* [34]. The maximum enrichment factors of Cd, Cu, Zn, and Pb in *S. salsa* can reach 64.32, 24.80, 10.30, and 14.20, respectively [33,35]. At present, there are many studies on the assessment of the heavy metal pollution risk in saline–alkali wetland soils and the heavy metal enrichment characteristics of salt-tolerant wetland plants [36,37]. But the changes in the physiological characteristics of *S. liaotungensis* seedlings exposed to heavy metal pollutants are still unclear.

This study tests the physiological changes in seedlings from dimorphic seeds of *S. liaotungensis* and their response to major heavy metal pollutants (Pb, Cd, Cu and Zn) in coastal marsh soils. In this study, our purpose is as follows: (1) to compare the difference in seedling growth to different heavy metal stress, (2) to analyze the physiological differences in seedlings from dimorphic seeds in response to different heavy metal stress, (3) to contrast the difference in the tolerance of seedlings between brown and black seeds at the same heavy metal stress. This study provides an important theoretical basis for the mechanism of heavy metal tolerance of *Suaeda liaotungensis*, which also makes *S. liaotungensis* a key actor in repairing coastal saline soil contaminated by heavy metals.

## 2. Materials and Methods

### 2.1. Plant Materials

*Suaeda liaotungensis* plants were harvested from saline–alkali soil in Yingchengzi Town, Dalian City, Liaoning Province, China at 121.36° E/38.99° N in November 2023. Dimorphic seeds of *S. liaotungensis* were screened and separated and then stored at 4 °C in a refrigerator for subsequent experiments.

### 2.2. Seedling Growth

To test the effect of heavy metals on the seedling growth of dimorphic seeds, 40 dimorphic seeds were, respectively, placed into a Petri dish with a layer of filter paper soaked in 10 mL different solutions: distilled water (control), Pb^2+^ solution (100, 200, 400, 800, and 1000 mg/L), Cd^2+^ solution (5, 10, 20, and 50 mg/L), Cu^2+^ solution (20, 50, 100, and 200 mg/L), and Zn^2+^ solution (50, 100, 200, and 500 mg/L), and then the seeds were placed in a plant incubator (GZX-300BS, CIMO, Shanghai, China). The culture condition was set at 20 °C for 12 h of light and 10 °C for 12 h of darkness. Germination was considered as a radicle protrusion ≥ 2 mm, and then germinated seeds were transferred to the same heavy metal solution and cultured for 15 days. The seedlings under different solutions treatment were photographed by a camera (Canon, Tokyo, Japan), and the root length and shoot length of seedlings were measured by Image J (version 1.44). Three biological replicates were performed. Following the published method, the lengths and tolerance indexes of 10 seedlings at tested concentrations were calculated for each replicate [38].

Root tolerance index (%) = (root length in different heavy metal treatments/root length in distilled water treatment) × 100.

Shoot tolerance index (%) = (shoot length in different heavy metal treatments/shoot length in distilled water treatment) × 100.

### 2.3. Detection of Physiological Indexes

The superoxide anion (O_2_^−.^), hydrogen peroxide (H_2_O_2_), and malondialdehyde (MDA) content were detected using the O_2_^−.^, H_2_O_2_, and MDA Content Test Kit (Solarbio, Beijing, China). O_2_^−.^ reacts with hydroxylamine hydrochloride to produce NO_2_^−.^. Under the action of *p*-aminobenzenesulfonamide and naphthalene ethylenediamine hydrochloride, the purplish-red azo compound was produced by NO_2_^−.^. The absorbance of 530 nm was measured by a spectrophotometer (Genova Nano, Jenway, Essex, UK). H_2_O_2_ and titanium sulfate produced a yellow peroxide complex with characteristic absorption at 415 nm. The absorbance values were measured at 415 nm wavelengths using the spectrophotometer (Genova Nano, Jenway, Essex, UK). MDA reacts with thiobarbituric acid to form brown-red 3,5,5-trimethyloxazol-2,4-dione under acidic and high-temperature conditions. Absorbance at 532 nm and 600 nm was measured by a spectrophotometer (Genova Nano, Jenway, Essex, UK).

The determination of superoxide dismutase (SOD), peroxidase (POD), and catalase (CAT) activities was performed using Song’s method [31]. Approximately 0.15 g seedlings for each sample were homogenized in phosphate buffer solution (PBS) (Sangon Biotech, Shanghai, China) and centrifuged at 4 °C for 30 min. The supernatant was designated as the crude extract of enzymes. Then, SOD activity was detected by the nitrotetrazolium blue (NBT) photochemical reduction method, and the absorbance of 560 nm was detected by a spectrophotometer (Genova Nano, Jenway, Essex, UK). POD activity was detected by the guaiacol method, and the absorbance of 470 nm was detected by a spectrophotometer (Genova Nano, Jenway, Essex, UK). CAT activity was detected by an ultraviolet spectrophotometry, and the absorbance of 240 nm was detected by a spectrophotometer (Genova Nano, Jenway, Essex, UK).

Soluble sugar content was measured using the anthrone colorimetry method. Adding 4 mL 80% (*v*/*v*) ethanol, 0.15 g seedlings were powdered using liquid nitrogen. After a water bath at 80 °C for 30 min, the extracted liquid was centrifuged at 4 °C, 12,000 rpm for 10 min. The supernatant was mixed with 0.9 mL 80% (*v*/*v*) ethanol, 0.5 mL 2% (*w*/*v*) anthrone ethyl acetate (Sangon Biotech, Shanghai, China), and 5 mL concentrated sulfuric acid (Kemiou, Tianjin, China). After 10 min of boiling in water, the absorbance value at a 620 nm wavelength was measured with a spectrophotometer (Genova Nano, Jenway, Essex, UK).

The proline content in seedlings was performed by the sulfosalicylic acid method [39], and the absorbance of 520 nm was detected by a spectrophotometer (Genova Nano, Jenway, Essex, UK).

### 2.4. Statistical Analysis

Statistical analysis was performed using SPSS 25.0 software. Variances in seedling growth and physiological traits under varying concentrations of heavy metals within the same seed type were analyzed using one-way ANOVA followed by an LSD test. Variances in physiological traits between brown and black seeds under the identical heavy metal treatments were analyzed using an independent sample *t*-test. Significant levels were set at *p* < 0.05. In addition, principal component analysis (PCA) and the logistic regression analysis were carried out utilizing the FactoMineR and Factoextra packages in R software version 4.2.2. The dplyr and tidyverse packages were used to process and analyze data. The ggplot2 (version 3.5.1) and GGally (version 2.2.1) packages were used to create the visualizations.

## 3. Results

### 3.1. Effect of Heavy Metals on Seedling Growth

**Figure 1:**
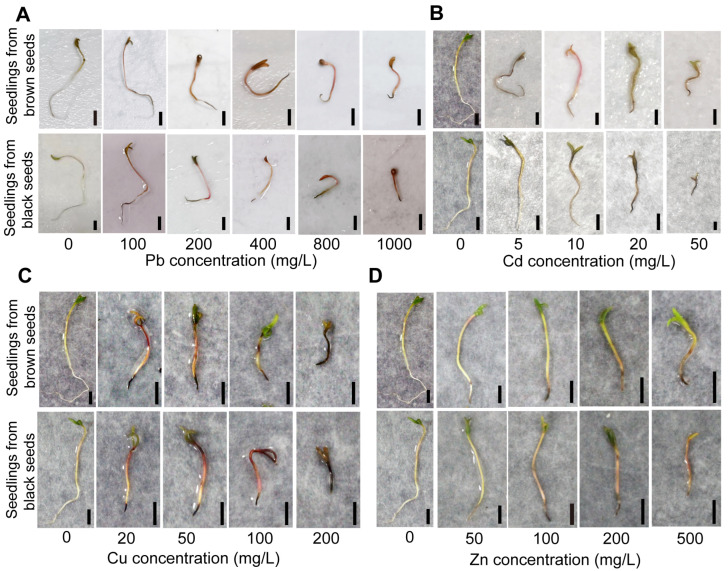
Phenotypes of seedlings from dimorphic seeds under heavy metal stress. Seedling growth of dimorphic seeds under Pb stress (**A**), Cd stress (**B**), Cu stress (**C**), and Zn stress (**D**). Bars = 5 mm.

Seedling growth was dramatically suppressed as concentrations of the tested heavy metals increased (Figure 1). Under Pb, Cd, and Cu stress, the seedlings from brown seeds had a considerably longer root length compared to those from black seeds, but the opposite trend appeared at 50 mg/L Zn (Table 1). Under Pb and Cd stress, the seedlings from black seeds had a longer shoot length compared to those from brown seeds, but the opposite trend appeared at 50 mg/L Cd (Table 1). The seedlings from brown seeds exhibited a markedly longer shoot length under Cu stress and at 500 mg/L Zn (Table 1). When the concentration of heavy metals increased, the tolerance indexes in seedlings typically displayed declining trends (Table 2). The seedlings derived from black seeds exhibited greater tolerance indexes compared to those from brown seeds under Pb stress, but the opposite trend was shown at 50 mg/L Cd and under Cu stress (Table 2). The seedlings from brown seeds displayed a lower root tolerance index under Zn stress, whereas seedlings had a higher shoot tolerance index at 500 mg/L Zn (Table 2).

### 3.2. Effect of Heavy Metals on ROS Levels in Seedlings

**Figure 2:**
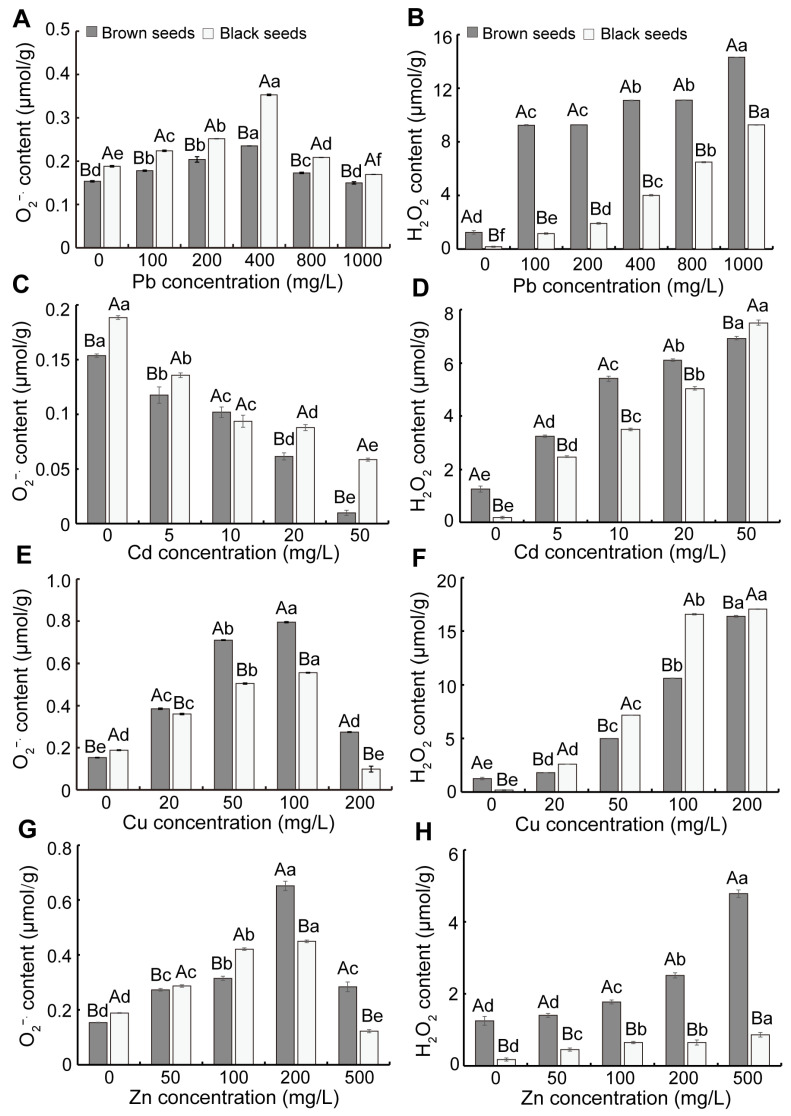
Effect of heavy metal stress on ROS content of seedlings of *S. liaotungensis*. O_2_^−.^ and H_2_O_2_ content in seedlings from dimorphic seeds under Pb stress (**A**,**B**), Cd stress (**C**,**D**), Cu stress (**E**,**F**), and Zn stress (**G**,**H**). Under the same heavy metal concentration, significant difference between brown and black seeds is represented by distinct uppercase letters (*p* < 0.05). Significant differences in O_2_^−.^ and H_2_O_2_ content among different concentrations of heavy metals for the same seed type are represented by distinct lowercase letters (*p* < 0.05).

**Figure 3:**
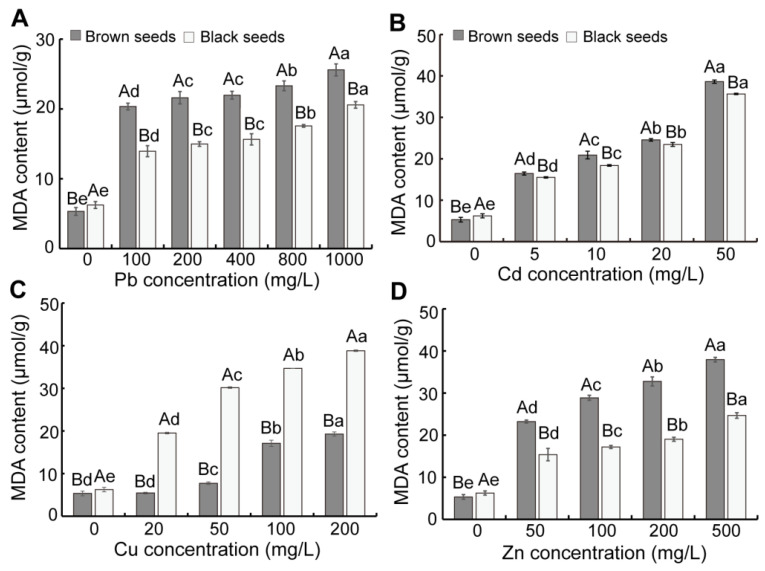
Effect of heavy metal stress on MDA content of seedlings of *S. liaotungensis*. MDA content in seedlings from dimorphic seeds under Pb stress (**A**), Cd stress (**B**), Cu stress (**C**), and Zn stress (**D**). Under the same heavy metal concentration, significant difference between brown and black seeds is represented by distinct uppercase letters (*p* < 0.05). Significant difference in MDA content among varying heavy metal concentrations for the same seed type is represented by distinct lowercase letters (*p* < 0.05).

As the Pb, Cu, and Zn concentration increased, the O_2_^−.^ content in seedlings from dimorphic seeds first ascended and subsequently declined, whereas the H_2_O_2_ content in them continuously increased (Figure 2). As the Cd concentration increased, the O_2_^−.^ content in seedlings from dimorphic seeds gradually decreased, whereas the H_2_O_2_ content in them significantly increased (Figure 2C,D). At the same Pb and Cd concentration, the seedlings from brown seeds exhibited lower O_2_^−.^ and higher H_2_O_2_ contents than those of black seeds (Figure 2A–D). Under Cu stress, the seedlings from brown seeds showed greater O_2_^−.^ content than that of black seeds, whereas the H_2_O_2_ content showed the opposite trend (Figure 2E,F). When treated with a high concentration of Zn, the seedlings from brown seeds showed greater O_2_^−.^ and H_2_O_2_ contents than those of black seeds (Figure 2G,H). In addition, as the concentration of heavy metals increased, the MDA content in seedlings gradually increased (Figure 3). Under Pb, Cd, and Zn stress, the seedlings derived from brown seeds exhibited markedly enhanced MDA content compared to that of black seeds, whereas there was an opposite trend under Cu stress (Figure 3).

### 3.3. Effect of Heavy Metals on Antioxidant Enzyme Activity in Seedlings

**Figure 4:**
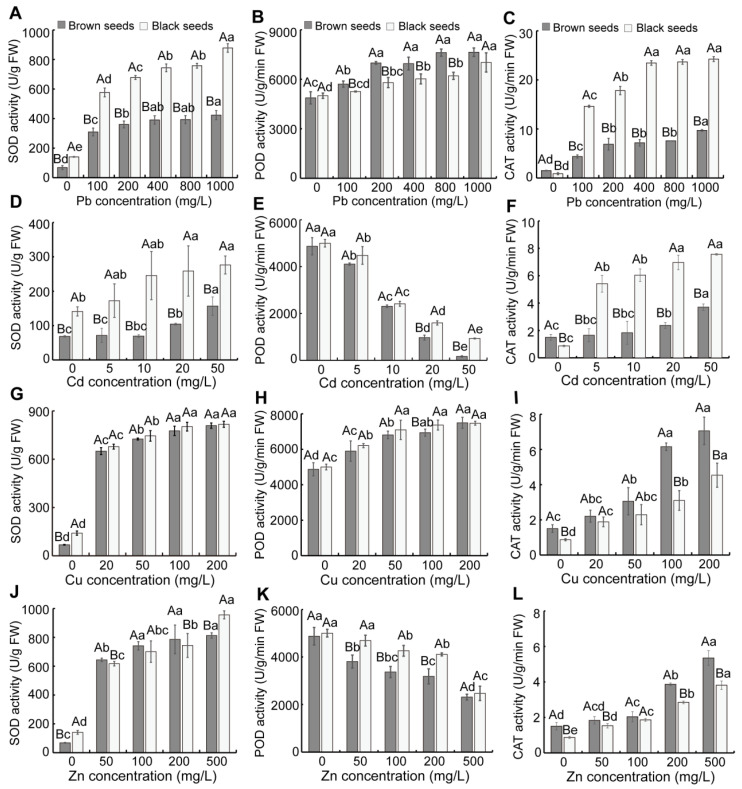
Effect of heavy metal stress on antioxidant enzyme activity of seedlings of *S. liaotungensis*. SOD, POD, and CAT activities in seedlings under Pb stress (**A**–**C**), Cd stress (**D**–**F**), Cu stress (**G**–**I**), and Zn stress (**J**–**L**). Under the same heavy metal concentration, significant difference between brown and black seeds is represented by distinct uppercase letters (*p* < 0.05). Significant differences in SOD, POD, and CAT activities among varying heavy metal concentrations for the same seed type are represented by distinct lowercase letters (*p* < 0.05).

As the concentration of Pb and Cu increased, the SOD, POD, and CAT activities of seedlings gradually ascended (Figure 4A–C,G–I). Under Cd and Zn stress, the SOD and CAT activities in seedlings from dimorphic seeds gradually increased, but the POD activity in them gradually decreased (Figure 4D–F,J–L). Under Pb stress, the seedlings from brown seeds had significantly lower SOD and CAT activities but increased POD activity compared to those of black seeds (Figure 4A–C). Under Cd stress, the seedlings from black seeds exhibited considerably higher SOD, POD, and CAT activities compared to those of brown seeds (Figure 4D–F). Under Cu stress, the seedlings from brown seeds exhibited considerably higher CAT activity than that of black seeds, but much lower POD activity (Figure 4H,I). Under Zn stress, the seedlings derived from brown seeds showed markedly enhanced CAT activity and decreased POD activity (Figure 4K,L).

### 3.4. Effect of Heavy Metals on Osmotic Regulating Substances in Seedlings

**Figure 5:**
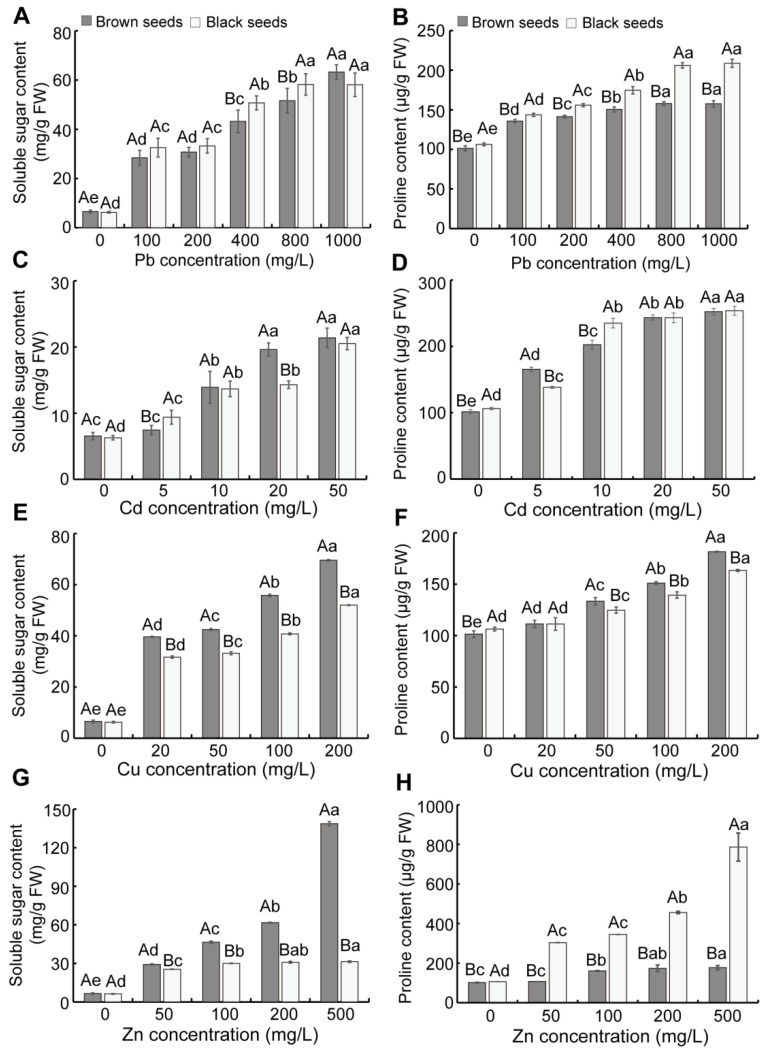
Effect of heavy metal stress on osmotic adjustment substances in seedlings from dimorphic seeds of *S. liaotungensis*. Soluble sugar and proline content of seedlings from dimorphic seeds under Pb stress (**A**,**B**), Cd stress (**C**,**D**), Cu stress (**E**,**F**), and Zn stress (**G**,**H**). Under the same heavy metal concentration, significant difference between brown and black seeds is represented by distinct uppercase letters (*p* < 0.05). Significant difference among varying heavy metal concentrations for the same seed type is represented by distinct lowercase letters (*p* < 0.05).

Soluble sugar, proline, and betaine play vital roles in stress tolerance. The accumulation of these substances helps to lower the osmotic potential of cells. As a result, we also determined the soluble sugar and proline content in seedlings. As the concentration of heavy metals increased, the soluble sugar and proline content in seedlings gradually ascended (Figure 5). Under Cu and Zn stress, the seedlings from brown seeds exhibited markedly enhanced soluble sugar content, whereas the seedlings from black seeds showed a similar trend under Pb stress (Figure 5A,E,G). Under Pb and Zn stress, the seedlings derived from black seeds exhibited markedly enhanced proline content, whereas those from brown seeds showed a similar trend under Cu stress (Figure 5B,F,H). When treated with a high concentration of Cd, the seedlings from brown seeds showed a greater soluble sugar content, while there was no difference in proline content (Figure 5C,D).

### 3.5. Principal Component Analysis, Correlation, and Regression Insights Under Heavy Metal Stress

**Figure 6:**
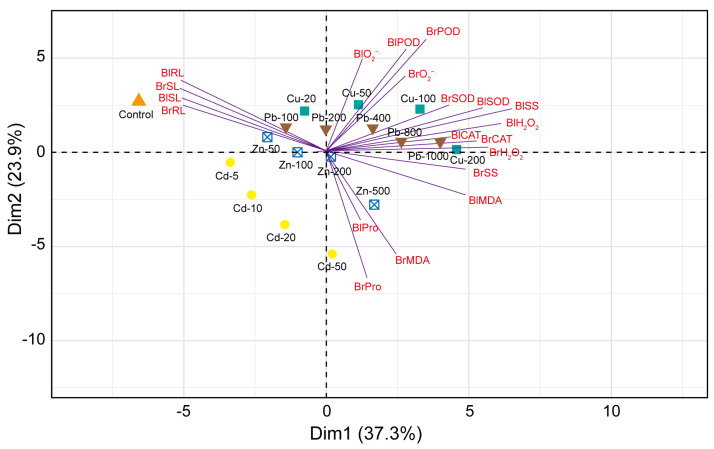
Principle component analysis (PCA) of physiological and biochemical traits of seedlings under heavy metal (Pb, Cd, Cu, and Zn) stress. Abbreviation: Br, brown seeds; Bl, black seeds; RL, root length; SL, shoot length; O_2_^−.^, superoxide anion radical; H_2_O_2_, hydrogen peroxide; MDA, malondialdehyde; SOD, superoxide dismutase; POD, peroxidase; CAT, catalase; SS, soluble sugar; Pro, proline.

To evaluate the relationship between the physiological traits of seedlings from dimorphic seeds under different concentrations of heavy metals (Pb, Cd, Cu, and Zn), we performed principal component analysis (PCA) (Figure 6). The PCA of five sample groups showed that the first two components accounted for 61.2% of the total variance, with the first principal component (PC1) accounting for 37.3% and the second principal component (PC2) for 23.9%. The PCA results exhibited that the O_2_^−.^, H_2_O_2_, MDA, antioxidant enzyme (SOD, POD, and CAT), soluble sugar (SS), and proline (Pro) contents belong to the PC1. However, the radicle length (RL) and shoot length (SL) belong to the PC2 (Figure 6). The PC1 had high positive values for BrO_2_^−.^ (0.120), BrH_2_O_2_ (0.280), BrMDA (0.111), BrSOD (0.212), BrPOD (0.172), BrCAT (0.302), BrSS (0.237), BrPro (0.070), BlO_2_^−.^ (0.062), BlH_2_O_2_ (0.259), BlMDA (0.242), BlSOD (0.292), BlPOD (0.164), BlCAT (0.150), BlSS (0.321), and BlPro (0.049). The PC2 had high negative values for BrRL (−0.272), BrSL (−0.258), BrRL (−0.263), and BrSL (−0.291). Therefore, the RL and SL were positively correlated with the control, Pb-treated (100 mg/L), Cu-treated (20 mg/L), and Zn-treated (50 and 100 mg/L) seedlings from dimorphic seeds. However, some physiological traits, including O_2_^−.^, H_2_O_2,_ MDA, SOD, POD, CAT, SS, and Pro, were negatively correlated with the control, Pb-treated (100 mg/L), Cu-treated (20 mg/L), and Zn-treated (50 and 100 mg/L) seedlings from dimorphic seeds.

**Figure 7:**
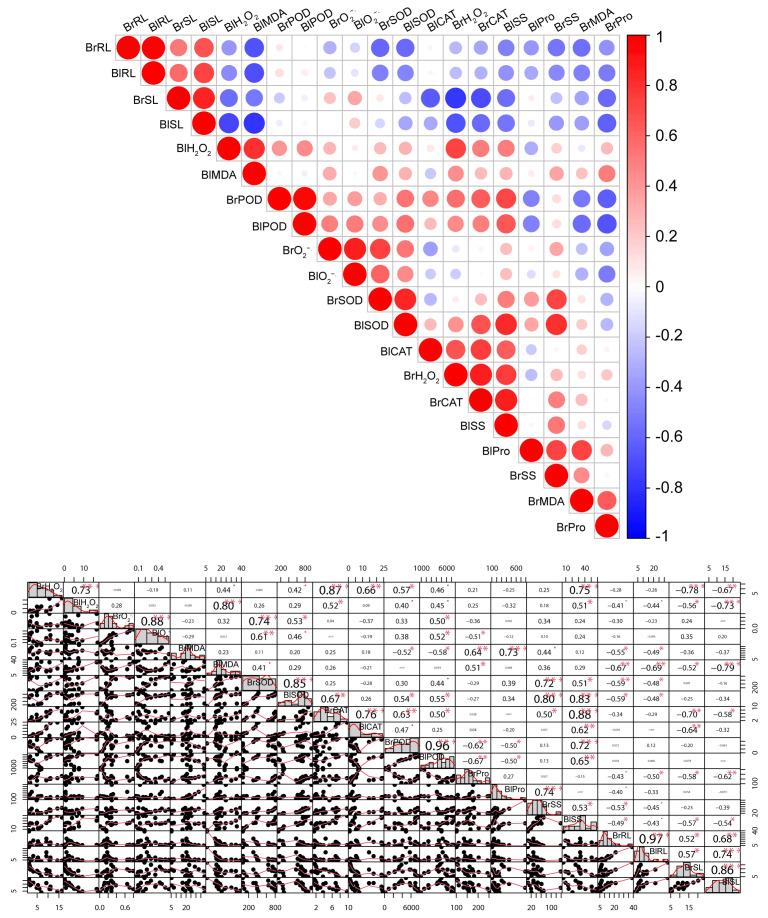
The correlation analysis of the physiological and biochemical traits of seedlings under heavy metal (Pb, Cd, Cu, and Zn) stress. Red and blue represent positive and negative correlations, respectively. Correlation values are significant at * *p*< 0.05, ** *p*< 0.01, and *** *p* < 0.001, respectively. The red dot represents 0.05 < *p* < 0.1. Abbreviation: Br, brown seeds; Bl, black seeds; RL, root length; SL, shoot length; O_2_^−.^, superoxide anion radical; H_2_O_2_, hydrogen peroxide; MDA, malondialdehyde; SOD, superoxide dismutase; POD, peroxidase; CAT, catalase; SS, soluble sugar; Pro, proline.

As shown in Figure 7, there was a correlation between the RL, SL, ROS levels; antioxidant enzymes system; and osmotic regulation system of seedlings from dimorphic seeds (Figure 7 and Table S1). The RL and SL had a negative correlation with other parameters including H_2_O_2_, MDA, SOD, CAT, SS, and Pro, whereas the RL and SL had a high positive correlation with each other (Figure 7). The BrRL showed a significant negative correlation with MDA, SOD, and SS, whereas the BlRL showed a strong negative correlation with MDA (Figure 7). The BrSL showed a strong negative correlation with H_2_O_2_, CAT, and Pro, whereas the BlSL showed a strong negative correlation with H_2_O_2_, MDA, and SS (Figure 7). In addition, POD and CAT exhibited a significant positive correlation with SS, whereas they showed a significant negative correlation with Pro. SOD showed a strong positive correlation with SS (Figure 7).

Linear regression analysis was performed on the physiological traits under different heavy metal stress (Figures S1–S4). Under Pb treatment, POD and soluble sugar showed strong and significant correlations in seedlings from brown seeds, whereas H_2_O_2_, POD, soluble sugar, and proline showed strong and significant correlations in the seedlings from black seeds. Under Cd treatment, O_2_^−.^, MDA, SOD, POD, and CAT exhibited strong and significant correlations in the seedlings from brown seeds, whereas H_2_O_2_, MDA, and soluble sugar exhibited strong and significant correlations in the seedlings from black seeds. Under Cu treatment, H_2_O_2_ and proline showed strong and significant correlations in seedlings from dimorphic seeds. Under Zn treatment, O_2_^−.^, H_2_O_2,_ and CAT showed strong and significant correlations in the seedlings from brown seeds, yet only CAT showed a strong and significant correlation in the seedlings from black seeds. Root and shoot length were strong correlations with the concentrations of heavy metals (Figure S5).

## 4. Discussion

A halophyte’s long-term adaptation to a saline environment is the cause of seed heteromorphism. In addition to its strong tolerance to saline–alkali soil, halophytes also have a great tolerance to heavy metal stress. The seedling phase is more susceptible to heavy metal stress than seed germination [40,41,42]. Heavy metal stress also inhibits seedling growth in *S. liaotungensis* (Figure 1). Under Cd stress, the root and coleoptile growth of seedlings were inhibited in *Sorghum bicolor* [43]. A significant reduction in radicle length was found with an increasing concentration of over 100 mg/L Cu and 500 mg/L Zn in *S. densiflora* [44]. In this study, the root and shoot lengths of seedlings were inhibited under all the tested Pb, Cd, Cu, and Zn stress (Table 1), which is consistent with the results of the seedlings of *Suaeda salsa* under heavy metal stress [45]. Heavy metals can inhibit cell division via denaturing nucleic acid or proteins in cells or affect cell elongation by inhibiting the formation of cytoskeletal proteins, which may be the reasons for the inhibition in seedling growth under heavy metal stress.

Plant roots are the earliest part to be exposed to heavy metals, and they accumulate many heavy metal ions, so roots are the most vulnerable part under heavy metal stress [46]. Our results also found that the degree of inhibition of the root length was greater than that of the shoot length under Cd, Cu, and Zn stress (Table 1). Meanwhile, seedlings from dimorphic seeds exhibited higher shoot tolerance indexes than root tolerance indexes under Pb, Cd, Cu, and Zn stress (Table 2), suggesting that the shoot of a seedling had stronger tolerance to Pb, Cd, Cu, and Zn stress than the root of one. Furthermore, the seedlings from black seeds were longer than those from brown seeds under Pb and Cd stress, whereas an opposite trend was observed under Cu stress (Table 1). These results demonstrate that the seedlings from black seeds have stronger tolerance to Pb and Cd stress, while those from brown seeds have stronger tolerance to Cu stress.

Earlier studies have demonstrated that ROS play a pivotal role at a signaling molecular level in modulating stress tolerance in plants [47,48]. Heavy metal stress, similarly to other abiotic stresses, induces elevated ROS levels [49,50]. Maintaining H_2_O_2_ content above a specific certain threshold level is crucial to prevent membrane lipid peroxidation [6]. In our study, the H_2_O_2_ and MDA content in seedlings was markedly ascended by Pb, Cd, Cu, and Zn treatment (Figure 2 and Figure 3). Similar results were observed in *Festuca arundinacea* and the halophytes *Suaeda fruticosa* and *Acanthus ilicifolius* [51,52,53]. The seedlings derived from brown seeds exhibited enhanced H_2_O_2_ and MDA contents under Pb, Cd, and Zn stress compared to those from black seeds. In contrast, a reverse pattern was observed under Cu stress (Figure 2), suggesting that brown seeds exhibited higher sensitivity to Pb, Cd, and Zn stress but showed greater tolerance to Cu stress.

When plants are exposed to heavy metals, they experience elevated levels of ROS, leading to oxidative damage [54,55,56]. To mitigate the oxidative damage induced by heavy metals, plants typically activate antioxidant defense mechanisms and modulate cellular metabolism to maintain cellular redox homeostasis [57,58,59,60]. In our study, the SOD and CAT activities in seedlings were markedly enhanced by Pb, Cd, Cu, and Zn treatment, whereas the POD activity in seedlings was ascended with increasing Pb and Cu concentrations (Figure 4), and similar findings were observed in the halophytes *Suaeda salsa*, *Atriplex atacamensis*, and *Salicornia europaea* [61,62,63]. In addition, the seedlings from brown seeds exhibited considerably higher POD activity compared to that of black seeds under Pb stress, whereas there was reduced POD activity under Cd, Cu, and Zn stress (Figure 4). The seedlings derived from black seeds displayed considerably enhanced CAT activity under Pb and Cd stress compared to those from brown seeds (Figure 4). In contrast, a reverse pattern was observed under Cu and Zn stress. The results suggest that POD and CAT have different roles in ROS scavenging under different heavy metal stress. In our study, we also found increased CAT activity in seedlings derived from brown seeds compared to in those from black seeds under Cu stress, which may be correlated with the decreased H_2_O_2_ and MDA content in seedlings from brown seeds.

To cope with environmental stress, plants accumulate diverse organic or inorganic substances to reduce osmotic potential and enhance cell water absorption [64]. Some osmotic adjustment substances including soluble sugar, proline, and betaine have the ability of maintaining intracellular ion balance, maintaining water absorption, removing metabolites, and alleviating oxidative damage [64,65]. The increased content of these substances enables the improvement in the plant’s stress resistance. In this study, the soluble sugar and proline content in seedlings steadily increased under heavy metal stress (Figure 5), which was consistent with the results of *Suaeda heteroptera* [66], which indicated that the intracellular osmotic balance was maintained by accumulating osmotic regulatory substances such as soluble sugar and proline, thereby enhancing the tolerance of seedlings to Pb, Cd, Cu, and Zn stress. Furthermore, the seedlings from black seeds exhibited higher soluble sugar and proline content compared to those from brown seeds under Pb stress, but the opposite tendency was observed under Cu stress (Figure 5), and these are reasons why the seedlings from black seeds are tolerant to Pb stress whereas the seedlings from brown seeds are tolerant to Cu stress. The seedlings from black seeds showed higher proline content compared to those from brown seeds under Zn stress (Figure 5H). Proline exhibits the capacity to mitigate ROS and suppress ROS-induced apoptosis during metal-induced oxidative stress. This was also the reason why the seedlings from black seeds had lower H_2_O_2_ and MDA content under Zn stress.

The diversity analysis of the physiological and biochemical traits of seedlings reflects the physiological response of seedlings to different heavy metal stress. In our study, the RL and SL had a negative correlation with some parameters such as H_2_O_2_, MDA, SOD, CAT, SS, and Pro (Figure 6 and Figure 7), suggesting that the content of H_2_O_2_, MDA, SOD, CAT, SS, and Pro increased with the decrease in the RL and SL. The RL and SL had a high positive correlation with each other. The uneven distribution of heavy metals in roots restricts the transport of nutrients to the shoot, consequently inhibiting shoot growth. The RL and SL of the seedlings from brown seeds and from black seeds were correlated with different physiological and biochemical indexes (Figure 7), which indicated that dimorphic seeds had different response mechanisms to heavy metal stress. Principle component analysis revealed significant differences in the response of seedlings to varying concentrations of heavy metals, leading to varying degrees of damage (Figure 6). Moreover, POD, CAT, SS, and Pro also exhibited a correlation, suggesting that as the concentration of heavy metals increased, POD, CAT, SS, and Pro contents increased to resist heavy metal stress.

Halophytes are naturally present in saline soils and can deal with abiotic stresses that occur in a natural environment [67]. These plants mainly accumulate large amounts of toxic ions such as Na^+^ and Cl^−^, making halophytes tolerant to other toxic metal ions as well [67]. Halophytes have evolved different strategies to withstand excess heavy metal ion toxicity including the immobilization and exclusion of heavy metals to reduce accumulation in plants, the chelation or compartmentalization of free metal ions in cells, the induction of oxidative stress defense systems, and the synthesis of osmotic substances and signaling molecules [67,68]. In our study, the accumulation of ROS in seedlings resulted in membrane lipid peroxidation under heavy metal (Pb, Cd, Cu, and Zn) stress, while the enhancement of SOD, POD, and CAT activities in seedlings reduced the damage of ROS on the cell membrane and maintained the stability of the cell membrane, thereby directly improving the tolerance of *S. liaotungensis* seedlings to heavy metal stress. *S. liaotungensis* seedlings increased the synthesis of osmoregulatory substances such as proline and soluble sugar under heavy metal (Pb, Cd, Cu, and Zn) stress, which alleviated ion toxicity by maintaining cell osmotic balance and indirectly enhanced tolerance to heavy metal stress. Heavy metal stress may activate the up-regulation of metal transporter genes which promote heavy metal ion efflux or vacuole compartmentalization [69]. The euhalophyte *Suaeda salsa* has a high ability for ion compartmentalization, such as Na+ and Cl−, in vacuoles [70]. Thus, vacuole metal sequestration should also be a strategy for the heavy metal tolerance of *S. liaotungensis*. In addition, heavy metal stress may regulate downstream gene expression in *S. liaotungensis* by activating endogenous hormones such as the ABA signaling pathway, thereby affecting the changes in physiological indicators (such as proline accumulation) and improving tolerance to heavy metal stress, but this needs to be further explored.

Compared with conventional approaches to remediate heavy metal-contaminated saline soil, phytoremediation is a more environmentally friendly technology [29]. Halophytes have high salt tolerance and the ability to accumulate heavy metals, making them potential plants for heavy metal-contaminated saline soil remediation [28,30]. The role of halophytes such as *Suaeda salsa*, *Suaeda glauca*, *Suaeda maritima,* and *Salicornia ramosissima* in heavy metal restoration has been reported [28,71,72,73]. Among them, the dry mass of the above-ground biomass of *S. salsa* under high salinity in the field is 1.8 times higher than that of the Cd-hyperaccumulator *Solanum nigrum* and 6.3 times higher than that of the Cd-hyperaccumulator *Viola baoshanensis* [35,74]. *S. salsa* can accumulate more Cr, Cu, Pb, and As when compared to other halophytes such as *Phragmites australis*, *Spartina alterniflora,* and *Typha orientalis* [34]. Meanwhile, *S. salsa* can accumulate Cd and Zn in the shoots and Cu and Pb in the roots [28]. These results demonstrate that *S. salsa* has a greater advantage in heavy metal-contaminated saline soil remediation. However, *S. liaongensis* and *S. salsa* belong to the same family and genus, and they are both distributed in the saline soil of the Liaohe Estuary wetland. In our study, *S. liaongensis* seedlings also had high tolerance to Pb, Cd, Cu, and Zn. The accumulation, migration, and transformation of heavy metals (Cu, Zn, Pb, and Cd) in *S. liaotungensis* may be similar to that of *S. salsa*, but this needs to be further explored. Different halophytes have a different tolerance and absorption capacity to different heavy metals. Therefore, it is also feasible to combine different halophytes to improve the removal ability of heavy metals. In addition, due to the structure and strong adsorption of biochar, the use of biochar in combination with halophytes will also receive more attention in coastal wetland soil remediation and improvement.

## 5. Conclusions

This study revealed that the seedling growth of dimorphic seeds was significantly inhibited under Pb, Cd, Cu, and Zn stress. The toxicity degree of Pb, Cd, Cu, and Zn to the seedlings of *S. liaotungensis* was Pb < Zn < Cu < Cd. The roots of seedlings from dimorphic seeds were more sensitive to heavy metal stress than the shoots. Some physiological and biochemical traits such as H_2_O_2_, MDA, SOD, CAT, soluble sugar, and proline showed an ascend trend, indicating that the osmotic regulation and antioxidant system of the seedlings were changed continuously in response to Pb, Cd, Cu, and Zn stress. In addition, the seedlings from black seeds have strong tolerance to Pb and Cd stress, while that of brown seeds have strong tolerance to Cu stress. This provides valuable data support for utilizing *S. liaotungensis* to repair heavy metal-contaminated saline soil.

## Figures and Tables

**Table 1:** Effect of heavy metal stress on seedling growth in *S. liaotungensis*.

Heavy Metal Concentration (mg/L)	Root Length (mm)		Shoot Length (mm)	
	Seedlings from Brown Seeds	Seedlings from Black Seeds	Seedlings from Brown Seeds	Seedlings from Black Seeds
Control	38.69 ± 4.33 Aa	33.24 ± 0.38 Ba	22.98 ± 0.73 Aa	23.66 ± 2.42 Aa
Pb				
100	21.46 ± 1.81 Ab	21.53 ± 0.42 Ab	14.08 ± 0.93 Bb	16.39 ± 1.54 Ab
200	13.53 ± 1.06 Ac	13.35 ± 1.23 Ac	8.97 ± 0.21 Bc	16.67 ± 0.58 Ab
400	12.03 ± 1.47 Acd	10.02 ± 0.79 Ad	7.49 ± 0.53 Bd	11.53 ± 0.40 Ac
800	8.53 ± 1.70 Ade	6.15 ± 0.46 Be	5.36 ± 0.90 Be	8.01 ± 1.10 Ad
1000	5.60 ± 0.20 Ae	3.89 ± 0.63 Bf	4.78 ± 0.64 Ae	4.59 ± 0.77 Ae
Cd				
5	16.94 ± 2.79 Ab	13.53 ± 2.15 Ab	11.69 ± 1.62 Bb	16.87 ± 2.59 Ab
10	14.81 ± 2.10 Ab	10.11 ± 1.33 Bc	11.09 ± 0.66 Bb	13.52 ± 0.51 Ac
20	8.36 ± 2.49 Ac	4.07 ± 0.83 Bd	9.41 ± 2.19 Ab	8.49 ± 1.28 Ad
50	4.13 ± 0.42 Ac	2.00 ± 0.18 Bd	5.19 ± 0.68 Ac	3.17 ± 0.37 Be
Cu				
20	10.91 ± 0.43 Ab	6.02 ± 0.36 Bb	13.92 ± 0.94 Ab	13.33 ± 0.79 Ab
50	6.64 ± 0.23 Ac	5.94 ± 0.58 Ab	12.49 ± 1.43 Abc	10.18 ± 0.90 Bc
100	6.37 ± 0.54 Ac	5.23 ± 0.79 Ab	12.07 ± 1.07 Ac	8.11 ± 0.27 Bc
200	4.35 ± 0.88 Ac	3.63 ± 0.17 Ac	5.72 ± 0.19 Ad	5.50 ± 0.45 Ad
Zn				
50	6.81 ± 0.37 Bb	7.68 ± 0.46 Ab	20.14 ± 1.43 Ab	19.86 ± 1.52 Ab
100	6.22 ± 0.40 Ab	6.18 ± 0.08 Ac	17.53 ± 0.65 Ac	17.25 ± 0.76 Ac
200	5.45 ± 0.12 Ab	5.52 ± 0.67 Acd	14.39 ± 0.75 Ad	15.14 ± 0.94 Ac
500	4.53 ± 0.44 Ab	4.96 ± 0.15 Ad	11.51 ± 0.17 Ae	9.83 ± 0.46 Bd

Under the same heavy metal concentration, significant difference between brown and black seeds is represented by distinct uppercase letters (*p* < 0.05). Significant difference among different concentrations of heavy metals for the same seed type is represented by distinct lowercase letters (*p* < 0.05). The data are presented as the means of three replicates ± SD.

**Table 2:** Root and shoot tolerance index in seedlings under heavy metal stress.

Heavy Metal Concentration (mg/L)	Root Tolerance Index		Shoot Tolerance Index	
	Seedlings from Brown Seeds	Seedlings from Black Seeds	Seedlings from Brown Seeds	Seedlings from Black Seeds
Control	100 ± 0.0 Aa	100 ± 0.0 Aa	100 ± 0.0 Aa	100 ± 0.0 Aa
Pb				
100	57.99 ± 5.90 Bb	68.47 ± 0.51 Ab	63.60 ± 2.38 Bb	70.71 ± 2.02 Ab
200	36.43 ± 3.83 Ac	41.90 ± 4.69 Ac	40.55 ± 0.67 Bc	72.09 ± 6.55 Ab
400	33.14 ± 7.60 Ac	32.63 ± 4.10 Ad	32.75 ± 2.62 Bd	50.16 ± 7.16 Ac
800	23.52 ± 7.37 Ad	19.32 ± 0.94 Ae	23.85 ± 3.92 Be	34.69 ± 1.79 Ad
1000	15.06 ± 1.43 Ad	12.46 ± 1.82 Af	21.08 ± 3.35 Ae	20.21 ± 5.14 Ae
Cd				
5	46.36 ± 8.15 Ab	43.01 ± 8.36 Ab	52.81 ± 4.25 Bb	72.80 ± 6.45 Ab
10	39.06 ± 4.87 Ab	30.99 ± 4.67 Ac	50.25 ± 3.87 Ab	58.96 ± 8.03 Ac
20	22.23 ± 5.84 Ac	13.16 ± 3.07 Bd	41.40 ± 8.54 Ac	37.51 ± 9.36 Ad
50	11.33 ± 1.81 Ad	6.38 ± 0.57 Bd	23.06 ± 3.11 Ad	13.95 ± 3.11 Be
Cu				
20	30.06 ± 2.88 Ab	19.33 ± 1.90 Bb	62.49 ± 2.55 Ab	57.67 ± 8.22 Ab
50	18.28 ± 2.02 Ac	18.70 ± 1.91 Ab	56.25 ± 6.16 Ac	44.47 ± 8.32 Ac
100	17.34 ± 2.19 Ac	16.97 ± 3.15 Ab	53.38 ± 2.81 Ac	35.38 ± 2.73 Bc
200	12.02 ± 3.88 Ad	11.68 ± 0.78 Ac	25.55 ± 2.26 Ad	24.28 ± 4.42 Ad
Zn				
50	18.49 ± 1.31 Bb	24.45 ± 0.75 Ab	90.29 ± 5.73 Ab	86.83 ± 15.54 Aab
100	17.05 ± 1.75 Bbc	19.72 ± 0.61 Ac	78.25 ± 5.40 Ac	75.08 ± 10.60 Abc
200	14.85 ± 1.77 Acd	17.61 ± 1.53 Ad	64.18 ± 1.31 Ad	65.94 ± 9.84 Ac
500	12.43 ± 2.81 Ad	15.66 ± 0.87 Ae	51.64 ± 1.55 Ae	42.60 ± 2.44 Bd

Under the same heavy metal concentration, significant difference between brown and black seeds is represented by distinct uppercase letters (*p* < 0.05). Significant difference among different concentrations of heavy metals for the same seed type is represented by distinct lowercase letters (*p* < 0.05).

## Data Availability

The data supporting the findings of this study can be obtained from the corresponding author upon reasonable request.

## Supplementary Materials

The following supplementary information can be downloaded at https://www.mdpi.com/article/10.3390/biology14030260/s1. Table S1. Principal component analysis of physiological traits of seedlings under different heavy metal stress. Figure S1. The correlation analysis of physiological traits of seedlings from dimorphic seeds treated with different concentrations of Pb. Figure S2. The correlation analysis of physiological traits of seedlings from dimorphic seeds treated with different concentrations of Cd. Figure S3. The correlation analysis of physiological traits of seedlings from dimorphic seeds treated with different concentrations of Cu. Figure S4. The correlation analysis of physiological traits of seedlings from dimorphic seeds treated with different concentrations of Zn. Figure S5. The correlation analysis between heavy metal concentration and seedling growth.
