# Supplementary material for: Physiological Adaptation to Different Heavy Metal Stress in Seedlings of Halophyte Suaeda liaotungensis
Source: Biology (Basel). 2025 Mar 5;14(3):260. doi: 10.3390/biology14030260 (PMC11940190; doi:10.3390/biology14030260)
Supplement: Supplementary file 1 [file biology-14-00260-s001.zip › biology-3488139-supplementary.pdf]

Table S1. Principal component analysis of physiological traits of seedlings under different heavy metals stress

Traits	PC1	PC2
**BrRL**	-0.27297131	-0.16546751
**BrSL**	-0.26362957	-0.18387655
**BlRL**	-0.25876324	-0.19307588
**BlSL**	-0.29189067	-0.19937885
**Br$O_2^{\cdot-}$**	0.12023779	-0.24126917
**BrH_2O_2**	0.28003282	-0.01160287
**BrMDA**	0.11172634	0.33847854
**BrSOD**	0.21238473	-0.14955690
**BrPOD**	0.17264060	-0.36648860
**BrCAT**	0.30200119	-0.05582216
**BrSS**	0.23763809	0.03380001
**BrPro**	0.07037811	0.41501825
**Bl$O_2^{\cdot-}$**	0.06226654	-0.29948344
**BlH_2O_2**	0.25929588	-0.01771681
**BlMDA**	0.24283753	0.14417881
**BlSOD**	0.29236311	-0.15112048
**BlPOD**	0.16477891	-0.39241421
**BlCAT**	0.15047228	-0.01633598
**BlSS**	0.32111215	-0.13748839
**BlPro**	0.04962662	0.22113049
**Eigen Values**	7.462147	4.783387
**Variance (%)**	37.31073	23.91694

Abbreviation: **Br**, brown seeds; **BL**, black seeds; **RL**, root length; **SL**, shoot length; **$O_2^{\cdot-}$**, superoxide anion radical; **H_2O_2**, hydrogen peroxide; **MDA**, malondialdehyde; **SOD**, superoxide dismutase; **POD**, peroxidase; **CAT**, catalase; **SS**, soluble sugar; **Pro**, proline.

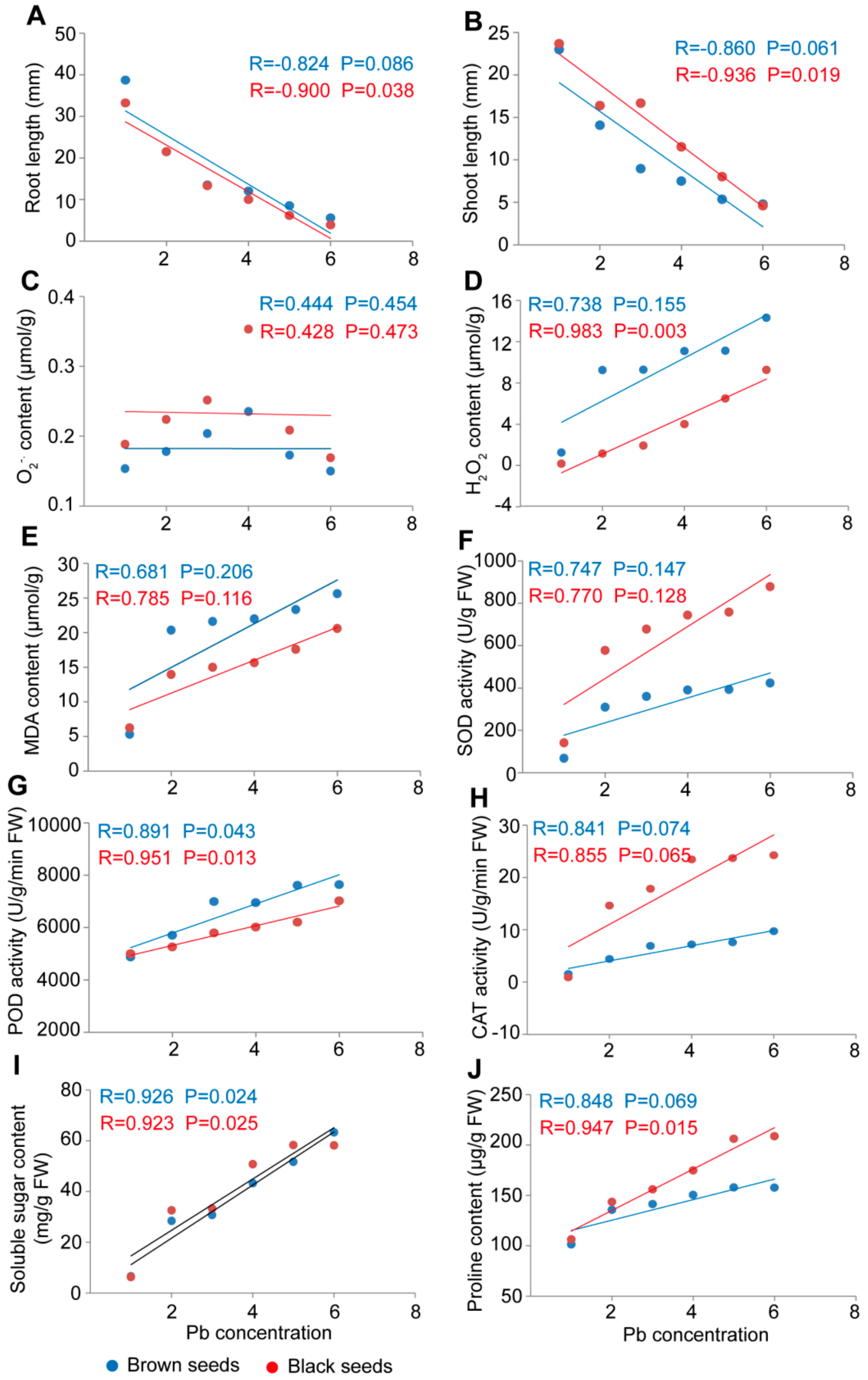

Figure S1. The correlation analysis of physiological traits of seedlings from dimorphic seeds treated with different concentrations of Pb. Correlation values are significant at $P < 0.05$.

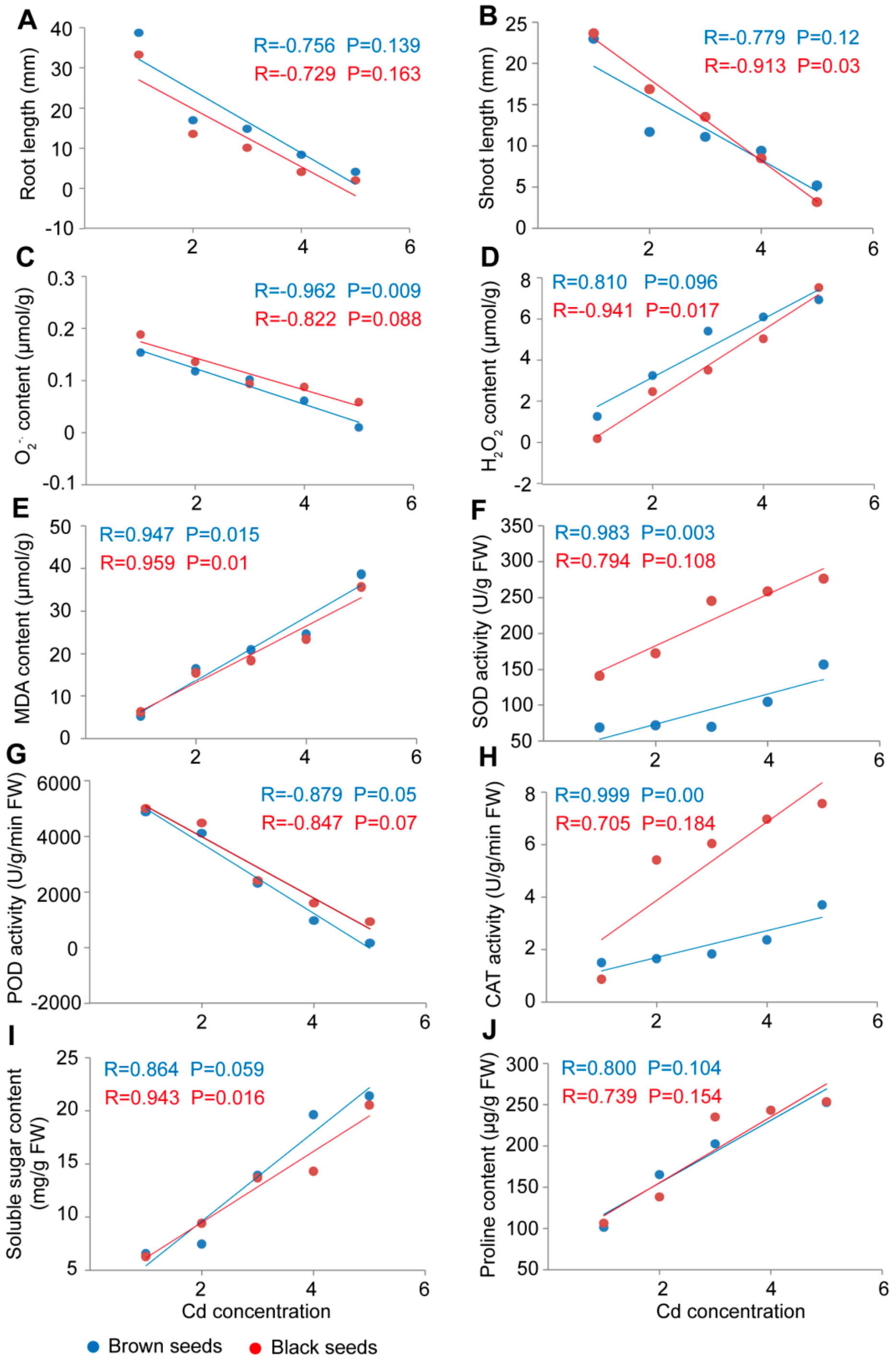

Figure S2. The correlation analysis of physiological traits of seedlings from dimorphic seeds treated with different concentrations of Cd. Correlation values are significant at $P < 0.05$.

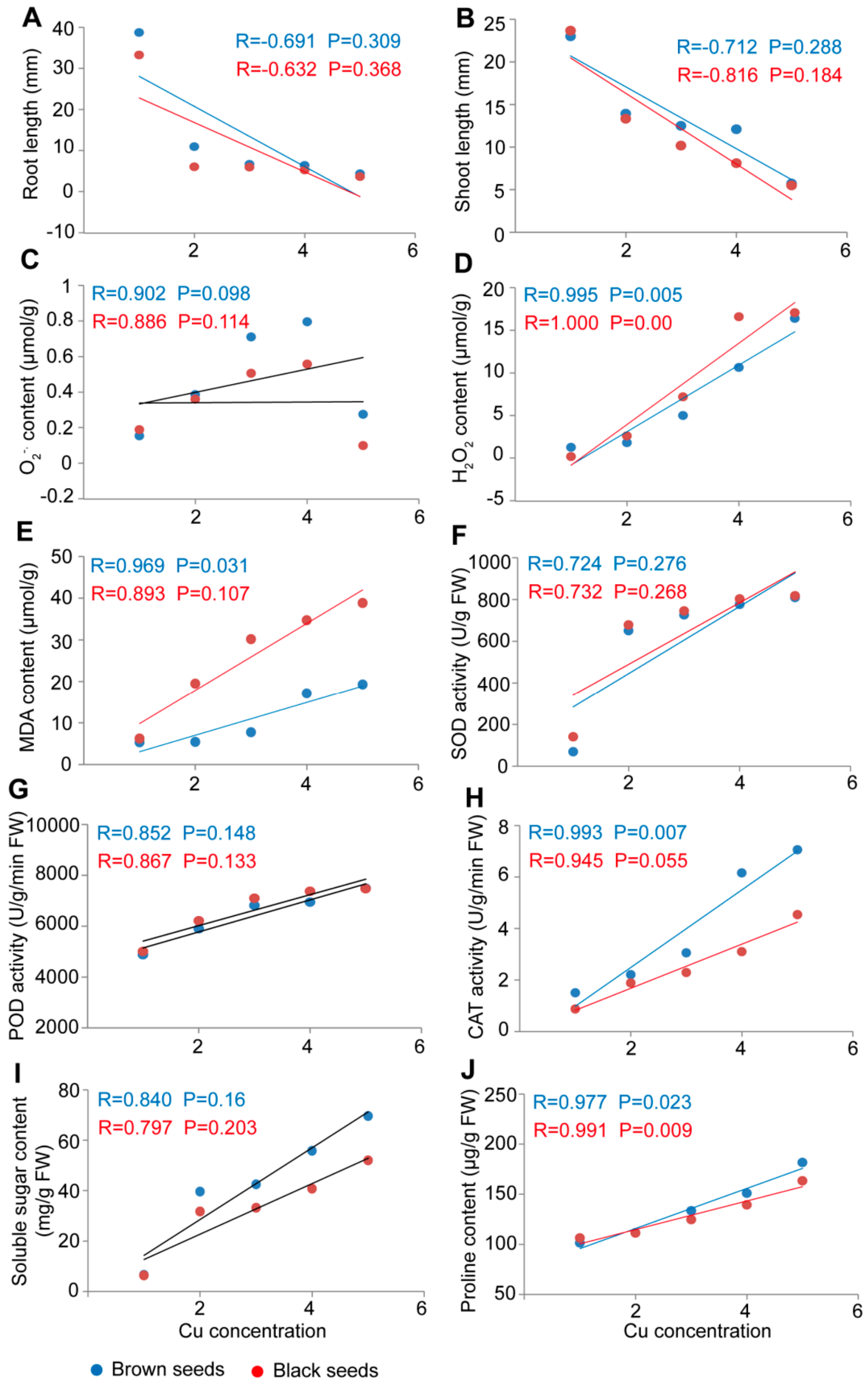

Figure S3. The correlation analysis of physiological traits of seedlings from dimorphic seeds treated with different concentrations of Cu. Correlation values are significant at $P < 0.05$.

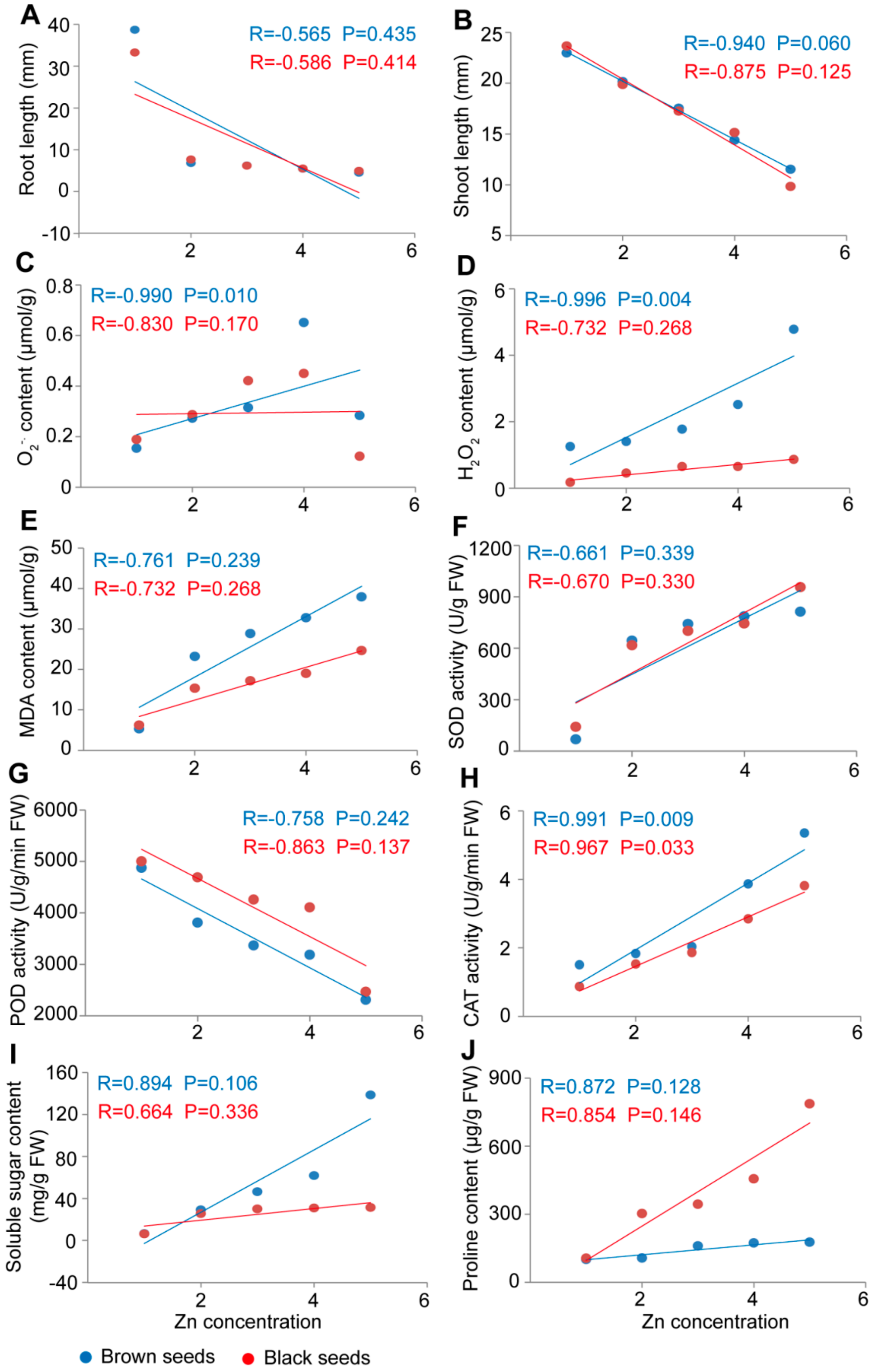

Figure S4. The correlation analysis of physiological traits of seedlings from dimorphic seeds treated with different concentrations of Zn. Correlation values are significant at $P < 0.05$.

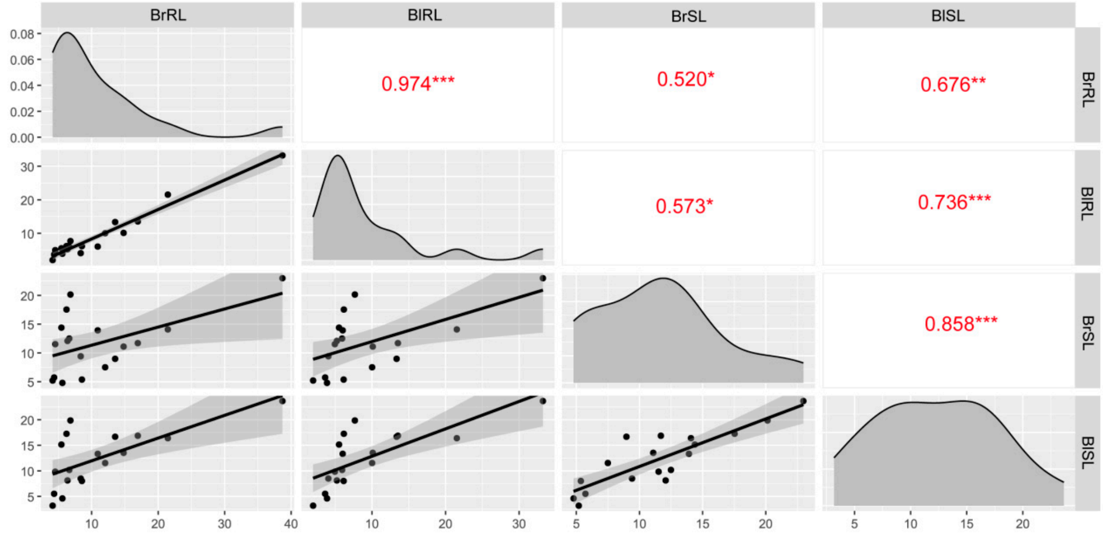

Figure S5. The correlation analysis between heavy metals concentration and seedling growth. Correlation values are significant at * P< 0.05, ** P< 0.01 and ***P < 0.001, respectively. The red font represents the correlation R value.
